# Reappraisal of IgG subclass deficiencies: a retrospective comparative cohort study

**DOI:** 10.3389/fimmu.2025.1552513

**Published:** 2025-04-17

**Authors:** Damla Dogru, Yagmur Dogru, Faranaz Atschekzei, Abdulwahab Elsayed, Natalia Dubrowinskaja, Diana Ernst, Torsten Witte, Vega Gödecke, Georgios Sogkas

**Affiliations:** ^1^ Department of Rheumatology and Immunology, Hannover Medical School, Hannover, Germany; ^2^ Cluster of Excellence RESIST (EXC 2155), Hannover Medical School, Hannover, Germany; ^3^ Center for Rare Diseases, Hannover Medical School, Hannover, Germany; ^4^ Department of Nephrology and Hypertension, Hannover Medical School, Hannover, Germany

**Keywords:** common variable immunodeficiency, IgG subclasses, IgG subclass deficiency, predominantly antibody deficiencies, bronchiectasis, arthritis, herpes zoster

## Abstract

**Objective:**

The aim of the present study was to investigate the clinical spectrum of IgG subclass deficiencies (IgGSDs) and assess the relative clinical significance of diagnosing each specific IgGSD disorder as compared to the common variable immunodeficiency (CVID).

**Methods:**

The clinical spectrum and immunological findings from 96 patients, diagnosed with diverse IgGSDs, were retrospectively evaluated. Specific IgGSDs were compared with each other and a cohort of 270 patients with CVID.

**Results:**

In comparison to CVID, recurrent lower respiratory tract infections (LRTIs) and bronchiectasis were rarer in IgGSDs, while recurrent mucocutaneous herpes simplex virus reactivations were more common. With respect to autoimmunity, IgGSDs were associated with arthritis, while autoimmune cytopenias were less frequently observed than in CVID. Among IgGSDs, herpes zoster was more common in IgG3SD. Arthritis was more prevalent in IgG1 + 3SD. Given its association with LRTI, splenomegaly, immune thrombocytopenic purpura, and the lower class-switched memory B-cell counts, IgG2 + 4SD is the IgGSD that rather resembles CVID.

**Conclusions:**

Comparative evaluation of phenotypes and treatments of patients with IgGSDs and CVID reveals distinct features, suggesting the differential clinical significance of diagnosing IgGSDs. The differential clinical expressions of IgGSDs highlight the need for studying each IgGSD separately in order to optimize disorder-specific follow-up procedures and prophylactic anti-infective measures.

## Introduction

Predominantly antibody deficiencies (PADs) are the most common subgroup of primary immunodeficiencies (PIDs) and comprise a heterogeneous group of disorders, whose main feature is ineffective humoral immunity (1, 2). In addition to immunological findings and recurrent infections, suggesting antibody failure, the clinical spectrum of PADs includes malignancies and variable manifestations of immune dysregulation, including autoimmunity, autoinflammation, and benign lymphoproliferation (3–5). Common variable immunodeficiency (CVID) is the most common symptomatic PAD (6). As is the case with the rest of PADs, CVID is differentiated from combined immunodeficiencies by the lack of clinical and immunological evidence of profound T-cell deficiency (7).

Immunoglobulin G (IgG) is the major out of the five classes of immunoglobulin and can be divided into four subclasses, i.e., IgG1, IgG2, IgG3, and IgG4, named in order of decreasing abundance in healthy adult human serum (8). The varying properties of the constant regions of IgG subclasses result in differential effector functions. IgG1 together with IgG3 antibodies display a higher potential to activate complement as well as a higher binding affinity to the Fcγ receptors. In addition to the differences in their concentration and molecular properties, IgG subclasses play different roles in immunity. For example, antibody responses to bacterial capsular polysaccharides are largely restricted to IgG2, while the production of IgG4 antibodies is commonly induced in the context of helminth and parasitic infections (9, 10).

IgG subclass deficiencies (IgGSDs) are characterized by reduced levels of one or more IgG subclasses, while total serum IgG levels remain normal (11–13). The clinical relevance of IgGSDs remains controversial. Associated clinical phenotypes range from asymptomatic states to severe or recurrent infections and varying manifestations of immune dysregulation (12–14). Autoimmunity has been reported to be the most common non-infectious manifestation of IgGSDs, affecting more than 40% of patients (15, 16). While some manifestations of IgGSDs, such as recurrent infections although encapsulated bacteria in the case of IgG2 subclass deficiency (IgG2SD), can be directly explained through the deficiency of the respective IgG subclass, others, such as autoimmunity, rather suggest a broader immune dysfunction.

Given the varying infectious and non-infectious manifestations of IgGSDs, in the present study, we evaluated the clinical spectrum of IgG in a German cohort of patients diagnosed with diverse IgGSD, according to current diagnostic criteria. Considering the controversial clinical relevance of IgGSDs, in contrast to most other PADs, the clinical features and treatments of patients with IgGSDs were compared to the ones of a cohort of patients with CVID.

## Material and methods

### Study cohort

This retrospective cohort study included adult patients (age ≥ 18 years) with PADs visiting the immunology outpatient clinic of the Hannover University Hospital. Clinical and laboratory data recorded between 10/2018 and 09/2024 from 270 patients with CVID and 96 patients with an IgGSD were analyzed retrospectively. All patients provided written informed consent, and the study was approved by the Ethics Committee of the Hannover Medical School (approval 11223_BO_K_2024). Diagnosis of primary immunodeficiency was based on the current European Society for Immunodeficiencies (ESID) diagnostic criteria (available at http://esid.org/Working-Parties/Registry/Diagnosis-criteria) (11).

### Clinical and immunological data

Clinical data were obtained from patients’ medical files. PAD-associated phenotypes were documented as described previously (5). They included recurrent upper and/or lower respiratory tract infections according to the national guidelines for the diagnosis of PIDs (https://register.awmf.org/de/leitlinien/detail/112-001) (17), bronchiectasis (computed tomography-confirmed), autoimmune cytopenias, such as autoimmune hemolytic anemia (AIHA), idiopathic thrombocytopenic purpura (ITP), organ-specific autoimmunity [including vitiligo, psoriasis, insulin-dependent diabetes mellitus (IDDM), thyroidopathies, atrophic gastritis, and arthritis], granulomatous disease, enteropathy, and malignancies. In particular, recurrent upper respiratory tract infections (URTIs) were defined as more than three upper respiratory tract infections (i.e., sinusitis, pharyngitis, or laryngitis), necessitating antibiotic treatment within 1 year for at least two consecutive years. Recurrent lower respiratory tract infections (LRTIs) were defined as more than three lower respiratory tract infections (i.e., bronchitis or pneumonia), necessitating antibiotic treatment within 1 year for at least two consecutive years and/or at least two radiologically confirmed cases of pneumonia within three consecutive years. Recurrent mucocutaneous herpes simplex virus (HSV) reactivation was defined as more than six reported episodes per year or at least two episodes per year for more than two consecutive years leading to systemic virostatic treatment or documentation of recurrent mucocutaneous herpes simplex virus reactivation leading to prophylactic virostatic treatment. Autoimmune inflammatory arthritis (hereinafter referred to as arthritis) was diagnosed by a rheumatologist based on clinical findings, after considering the diagnosis of septic or crystal arthritis, and was further classified according to relevant criteria [i.e., the American College of Rheumatology (ACR), European League Against Rheumatism (EULAR), Assessment of SpondyloArthritis International Society (ASAS), Classification Criteria for Psoriatic Arthritis (CASPAR), or International League of Associations for Rheumatology (ILAR) classification criteria], as performed previously (3). Interstitial lung disease (ILD) was diagnosed based on typical computed tomography scan findings, in the absence of evidence for an infectious or alternative cause. Splenomegaly was defined as spleen enlargement ≥11 cm on palpation or ultrasound, including previous splenectomy of an enlarged spleen. Lymphadenopathy was detected on palpation, ultrasound, computed tomography, or magnetic resonance imaging. Granulomatous disease was defined as at least one biopsy-proven unexplained granuloma, excluding Crohn’s disease-associated granulomas. Enteropathy included all cases of biopsy-proven non-infectious inflammatory bowel disease (IBD) (ulcerative colitis and Crohn’s disease), celiac disease, lymphocytic infiltration of the intraepithelial mucous, the lamina propria, and/or the submucosa as well as patients with chronic idiopathic diarrhea. Malignancies included hematologic and all other forms of cancer. Immunoglobulin replacement is licensed for primary antibody deficiencies and was considered, according to national guidelines, also for patients with IgGSDs, in the case of a persistent pathological susceptibility to infection despite prophylactic antibiotics (https://register.awmf.org/assets/guidelines/189-001l_S3_Therapie-primaerer-Antikoerpermangelerkrankungen-2019-05-verlaengert.pdf) (18).

Immunoglobulin levels, including IgG subclasses, were documented prior to the introduction of immunoglobulin replacement, while all considered immunoglobulin and lymphocyte count values were evaluated at least 6 months apart from treatment with immunosuppressive medications. Phenotypic analyses of lymphocytes from peripheral blood were performed as described previously (19). Briefly, peripheral blood mononuclear cells (PBMCs) were isolated from peripheral whole blood collected in sterile lithium heparin tubes. Phenotypic analyses were performed as multicolor immunofluorescence of PBMCs, using directly labeled monoclonal antibodies. A total of 1 × 10^5^ to 2 × 10^6^ cells/well were incubated with murine monoclonal antibodies against the appropriate antigens at an optimal dilution for 20 min at 4°C. Non-specific binding was eliminated by mixing the samples with a 1:5 solution of a commercial human IgG (Octagam, Octapharma, Lachen, Switzerland). Samples were washed three times in phosphate-buffered saline (PBS)/ bovine serum albumin (BSA), and at least 10^4^ cells per appropriate gate were analyzed. The following antibodies (all purchased from BioLegend, San Diego, CA, USA, if not otherwise stated) were used for this study: CD3 PerCP (BD Pharmingen, San Diego, CA, USA), CD3 PE-Cy7, CD3 BUV563 (BD Biosciences, San Jose, CA, USA), abTCR BU711, abTCR APC, CD4 APC-Cy7, CD4 PerCP, CD8 PE, CD16 FITC, CD19 BV510, CD21 PE, CD24 FITC, CD27 BUV661 (BD Biosciences), CD27 FITC, CD28 PE-Cy5, CD28 APC, CD31 FITC, CD38 PECy7, CD45 APC-Cy7, CD45RO PE-Dazzle 594, CD45RO BV421, CD45 BV785, CD45RA V500, CD56 BV421, CCR7 PE, IgD PE, and IgM Alexa Fluor 647. Each flow cytometric analysis was controlled with appropriate isotype-matched antibodies. CD19+ cells in the lymphocyte gate were subdivided into the following subsets: naïve B cells (IgD+, IgM+, and CD27−), IgM+ memory B cells (CD27+, IgD+, IgM+), class-switched B cells (CD27+, IgM−, and IgD−), plasmablasts (CD19+dim, CD27++, and CD38++), transitional B cells (IgM++, CD38++, and CD24+), and CD21 low B cells (CD38 low and CD21 low). NK cells were gated as CD56+CD3− cells. CD3+ cells in the lymphocyte gate were subdivided into the following subsets: CD4+ T cells, CD8+ T cells, naïve CD4+ T cells (CD4+ and CD45RA+), CD4^+^ memory T cells (CD4+ and CD45RO+), and follicular-like CD4+ T cells (CD4+, CD45RO+, and CCR5+).

### Statistical analysis

For statistical calculation, GraphPad prism 9 (GraphPad, La Jolla, CA, USA) was used. Descriptive statistics are reported as median and interquartile range (IQR) in the case of continuous variables and as counts and percentages for dichotomous variables. Categorical variables were compared by Yate’s continuity corrected chi-squared test, which was employed to compare CVID with IgGSDs as well as each IgGSD with the rest. Differences in lymphocyte counts between patients with IgGSDs and CVID were evaluated using the Mann–Whitney test, while for the comparison of CVID with distinct IgGSDs, ordinary one-way ANOVA and Tukey’s multiple comparison test were employed.

## Results

### Patients’ characteristics and overall mortality rate

**Figure 1:**
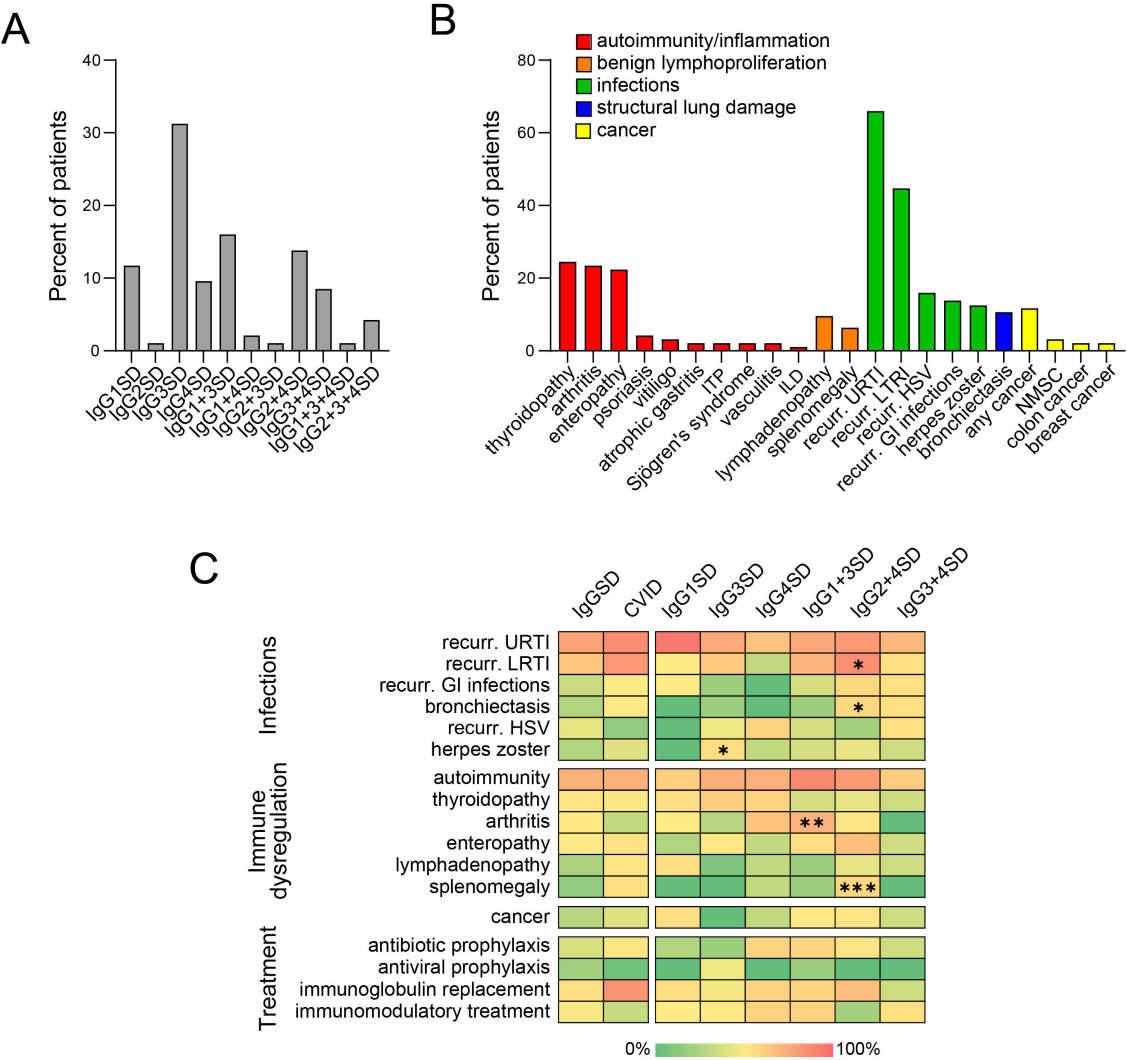
Diverse IgG subclass deficiencies (IgGSDs) **(A)** and associated clinical manifestations **(B)** in 96 patients. Heatmaps **(C)** displaying the relative frequencies of diverse clinical manifestations and treatments in patients with common variable immunodeficiency (CVID) as compared to cumulative group of patients with IgGSDs (left) as well as across the distinct IgGSD (right). Significant differences are marked with “*” (*p* < 0.05 *; *p* < 0.01 **; *p* < 0.001 ***, GI, gastrointestinal; HSV, herpes simplex virus; ILD, interstitial lung disease; ITP, immune thrombocytopenic purpura; LRTI, lower respiratory tract infections; NMSC, non-melanoma skin cancer; URTI, upper respiratory tract infections).

Among IgGSDs, IgG3SD was the most common diagnosis (30/96, 31.3%), followed by IgG1 + 3SD (15/96, 15.6%) and IgG2 + 4SD (13/96, 13.5%) (**Figure 1**). In contrast, the isolated reduction in IgG2 levels (i.e., IgG2SD) and the combined reduction in IgG2 and IgG3 levels (i.e., IgG2 + 3SD) were the rarest IgGSDs (2/96, 2.08% in the case of each disorder). There was a female predominance in IgGSDs as compared to CVID (71/96 vs. 160/270, *p* = 0.0147) (**Table 1**). Patients with CVID and IgGSDs were of similar age at analysis [mean age 47.7 ± 16.2 (in IgGSDs) vs. 46.4 ± 17.1 (in CVID), *p* = 0.5651]. However, patients with CVID were approximately 10 years younger at diagnosis as compared to IgGSDs [mean age at diagnosis 43.8 ± 16.9 (in IgGSDs) vs. 33.7 ± 16.3 (in CVID), *p* < 0.0001].

**Table 1:** Characteristics of studied patients with IgGSDs and CVID.

	IgGSD (N = 96)	CVID (N = 270)	OR	95% CI	*p*-Value^1^
Male sex, no. (%)	25 (26.04)	110 (40.74)	0.5122	0.3008–0.8618	0.0147 (*)
Median age, years (IQR)	47.35 (34.93–57.65)	46.95 (32.4–59.54)	n.a.	n.a.	0.5651 (ns)
Median age at diagnosis of IgGSD/CVID, years (IQR)	43.6 (30.5–55.8)	32.73 (21.1–44.67)	n.a.	n.a.	<0.0001 (****)
Recurrent URTI, no. (%)	62 (64.58)	213 (78.89)	0.488	0.2959–0.8256	0.0081 (**)
Recurrent LRTI, no. (%)	42 (43.75)	195 (72.22)	0.2991	0.1878–0.4834	<0.0001 (****)
Bronchiectasis, no. (%)	19 (10.42)	61 (22.59)	0.3984	0.1965–0.8163	0.0146 (*)
Recurrent GI infections, no. (%)	13 (13.54)	59 (21.85)	0.5601	0.2888–1.053	0.1074 (ns)
Recurrent mucocutaneous herpes simplex infections, no. (%)	17 (17.71)	17 (6.3)	3.203	1.603–6.373	0.0019 (**)
Herpes zoster, no. (%)	12 (12.5)	45 (16.67)	0.7143	0.3729–1.428	0.4219 (ns)
Thyroidopathy, no. (%)	23 (23.96)	54 (20)	1.26	0.7153–2.15	0.5019 (ns)
Arthritis, no. (%)	22 (22.92)	35 (12.96)	1.996	1.113–3.564	0.0319 (*)
ITP, no. (%)	2 (2.08)	45 (16.67)	0.1064	0.02497–0.4044	0.0005 (***)
AIHA, no. (%)	0 (0)	21 (7.78)	0	0–0.439	0.0105 (*)
ILD, no. (%)	1 (1.04)	34 (12.59)	0.07307	0.007064–0.4283	0.0019 (**)
Enteropathy, no. (%)	21 (21.88)	72 (26.67)	0.77	0.4438–1.342	0.4297 (ns)
Psoriasis, no. (%)	4 (4.17)	16 (5.93)	0.6902	0.2452–1.944	0.6966 (ns)
Vitiligo, no. (%)	3 (3.13)	9 (3.33)	0.9355	0.2678–3.498	0.8141 (ns)
Lymphadenopathy, no. (%)	9 (9.38)	65 (24.07)	0.3263	0.1571–0.6603	0.0034 (**)
Splenomegaly, no. (%)	6 (6.25)	75 (27.78)	0.1733	0.07855–0.3912	<0.0001 (****)
Cancer, no. (%)	11 (11.46)	45 (16.67)	0.6471	0.3184–1.27	0.2926 (ns)

AIHA, autoimmune hemolytic anemia; CI, confidence interval; CVID, common variable immunodeficiency; GI, gastrointestinal; IgGSD, IgG subclass deficiency; ILD, interstitial lung disease; ITP, immune thrombocytopenic purpura; LRTI, lower respiratory tract infection; N, total number; n.a., not applicable; no., number; OR, odds ratio; URTI, upper respiratory tract infection; ns, non-significant.

^1^ *p* < 0.05, *; *p* < 0.01, **; *p* < 0.001, ***; *p* < 0.0001, ****.

A single patient with IgGSD died. The cause of death was unrelated to IgGSD (sudden cardiac arrest). Among CVID patients, 11 (4.1%) patients died. Causes of death were documented in the case of 10/11 patients and related to liver failure (four patients), epithelial cancers (two patients), sepsis (two patients), non-Hodgkin lymphoma (one patient), and ILD (one patient). Overall, the difference in mortality rate was not significant (1/96 vs. 11/270, *p* = 0.2716), although death-related to disease-related manifestations, such as infectious complications, ILD, or hepatopathy, was documented only among CVID patients.

### Clinical phenotypes of IgGSDs in comparison to CVID

Common clinical manifestations of IgGSDs included infections, especially recurrent upper and lower respiratory tract infections (62/96, 64.6%, and 42/96, 43.8%, respectively), and autoimmune disorders, more commonly thyroiditis (23/96, 24%) and arthritis (22/96, 22.9%) (**Figure 1**). Arthritis could be commonly classified as spondyloarthritis in half of the patients (11/22, **Supplementary Figure 1**). Comparison of IgGSD with CVID with respect to infectious manifestations revealed that both upper and lower respiratory tract infections as well as bronchiectasis were less common in the case of IgGSDs (URTI: 62/96 vs. 213/270, *p* = 0.0081; LRTI: 42/96 vs. 195/270, *p* < 0.0001; bronchiectasis: 19/96 vs. 61/270, *p* = 0.0146) (**Table 1**, **Figure 1**). In contrast, recurrent mucocutaneous HSV reactivations were more prevalent in IgGSDs (17/96 vs. 17/270, *p* = 0.0019). The localization of mucocutaneous herpes was similar in IgGSDs and CVID (IgGSD: perioral: 15/17, genital 4/17, nasal 1/17; CVID: perioral: 12/17, genital 5/17). Among patients with IgGSDs, one male patient with IgG1 + 3+4SD had, in addition to recurrent perioral herpes, at least three episodes of herpes keratitis. An additional female patient with IgG1 + 3 deficiency and recurrent nasal and perioral herpes was diagnosed with herpes simplex encephalitis. The diagnosis of a specific disorder (i.e., IgG1SD, IgG3SD, IgG4SD, IgG1 + 3SD, IgG2 + 4SD, and IgG3 + 4D) as well as the presence of reduced levels of any of the four IgG subclasses did not correlate with recurrent mucocutaneous HSV reactivations (**Supplementary Table 1**). Further, IgGSD deficiency patients with and without a history of recurrent mucocutaneous HSV reactivations display no significant differences in studied lymphocyte subsets (**Supplementary Table 2**). With respect to manifestations of immune dysregulation, autoimmune cytopenias, in particular ITP and AIHA as well as manifestations of benign lymphoproliferation, i.e., lymphadenopathy and splenomegaly, were significantly less frequent in IgGSDs as compared to CVID (ITP: 2/96 vs. 45/270, *p* = 0.0005; AIHA: 0/96 vs. 21/270, *p* = 0.0105; lymphadenopathy: 9/96 vs. 65/270, *p* = 0.0034; splenomegaly: 6/25 vs. 75/270, *p* = 0.1733). ILD was also considerably rarer in the case of IgGSDs and was diagnosed in a single patient with IgG4SD (1/96 vs. 34/270, *p* = 0.0019). However, arthritis had been more frequently diagnosed in patients with IgGSDs (22/96 vs. 35/270, *p* = 0.0319). Regarding anti-infective prophylactic treatment, patients with IgGSDs were more commonly receiving antiviral prophylaxis (8/96 vs. 5/270, *p* = 0.0086), while patients with CVID were more commonly treated with immunoglobulin replacement (27/96 vs. 202/270, *p* < 0.0001) (**Table 2**). The latter comes in line with the above-discussed differences in the prevalence of herpes simplex virus reactivations and respiratory tract infections and suggests the differential clinical significance of infectious manifestations in IgGSD and CVID.

**Table 2:** Treatment of IgGSDs as compared to CVID.

	IgGSD (N = 96)	CVID (N = 270)	OR	95% CI	*p*-Value^1^
Immunoglobulin replacement, no. (%)	27 (28.13)	202 (74.81)	0.1317	0.07706–0.2254	<0.0001 (****)
Antibiotic prophylaxis, no. (%)	15 (15.63)	66 (24.44)	0.5724	0.3011–1.06	0.1 (ns)
Antiviral prophylaxis, no. (%)	8 (8.33)	5 (1.85)	4.818	1.477–13.3	0.0086 (**)
Immunomodulatory treatment^2^, no. (%)	19 (19.79)	36 (13.33)	1.604	0.8685–2.938	0.1755 (ns)

CI, confidence interval; CVID, common variable immunodeficiency; IgGSD, IgG subclass deficiency; N, total number; no., number; OR, odds ratio; ns, non-significant.

^1^ *p* < 0.01, **; *p* < 0.0001, ****.

^2^ These included systemic glucocorticoid treatment as well as disease-modifying antirheumatic drugs (DMARDs), i.e., conventional synthetic, targeted synthetic, and biological DMARDs.

### Comparison of the clinical phenotypes of different IgGSDs

Comparison between relatively common IgGSDs with respect to infectious manifestations revealed that recurrent lower respiratory tract infections were significantly more common among patients with IgG2 + 4SD [10/13 (76.9%) vs. 32/83 (38.6%), *p* = 0.0219, OR: 5.31, 95% CI: 1.33–18.76] (**Figure 1**). The same subgroup of patients displayed a significantly higher rate of bronchiectasis [4/13 (30.8%) vs. 6/83 (7.2%), *p* = 0.0361, OR: 5.7, 95% CI: 1.54–21.46]. It is noteworthy that the prevalence of bronchiectasis in the subgroup of patients with IgG2 + 4SD was similar to the one in patients with CVID [4/14 (30.8%) vs. 61/270 (22.6%), respectively, *p* = 0.7285, OR: 1.52, 95% CI: 0.5–5]. The latter results suggest the infectious etiology of bronchiectasis in patients with IgG2 + 4SD. Herpes zoster had been most commonly diagnosed among patients with IgG3SD [8/30 (26.7%) vs. 4/66 (6.1%), *p* = 0.0125, OR: 5.64, 95% CI: 1.67–17.79]. Given the fact that currently, the recombinant zoster vaccine (Shingrix) is indicated from the age of 50 years (20), we evaluated the frequency of herpes zoster manifesting prior to that age, as a higher risk for “early-onset” herpes zoster in the subgroup of patients with IgG3SD could suggest the need for earlier vaccination. However, herpes zoster prior to the age of 50 years was documented in the case of four patients with IgG3SD, yielding a quote of patients not significantly different from the rest of IgGSDs [4/30 (13.3%) vs. 5/66 (7.6%), *p* = 0.6035, OR: 1.88, 95% CI: 0.54–7]. Regarding anti-infective prophylactic treatment, no significant differences were observed across the different IgGSDs. However, given the higher frequency of herpes zoster in IgG3SD, it is noteworthy that out of eight patients receiving prophylactic antiviral treatment, the majority (i.e., 5/8) were diagnosed with IgG3SD. Regarding immune dysregulation, arthritis was significantly more common in the subgroup of patients with IgG1 + 3SD [8/15 (53.3%) vs. 14/81 (17.3%), *p* = 0.0066, OR: 5.47, 95% CI: 1.81–17.02], while ITP and splenomegaly were more prevalent in the case of patients with IgG2 + 4SD [ITP: 2/13 (15.4%) vs. 0/83 (0%), *p* = 0.0103, splenomegaly: 4/13 (30.8%) vs. 2/83 (2.4%), *p* = 0.0009, OR = 18, 95% CI: 3.53–98.67] (**Figure 1**).

### Immunological findings of patients with IgGSDs

**Figure 2:**
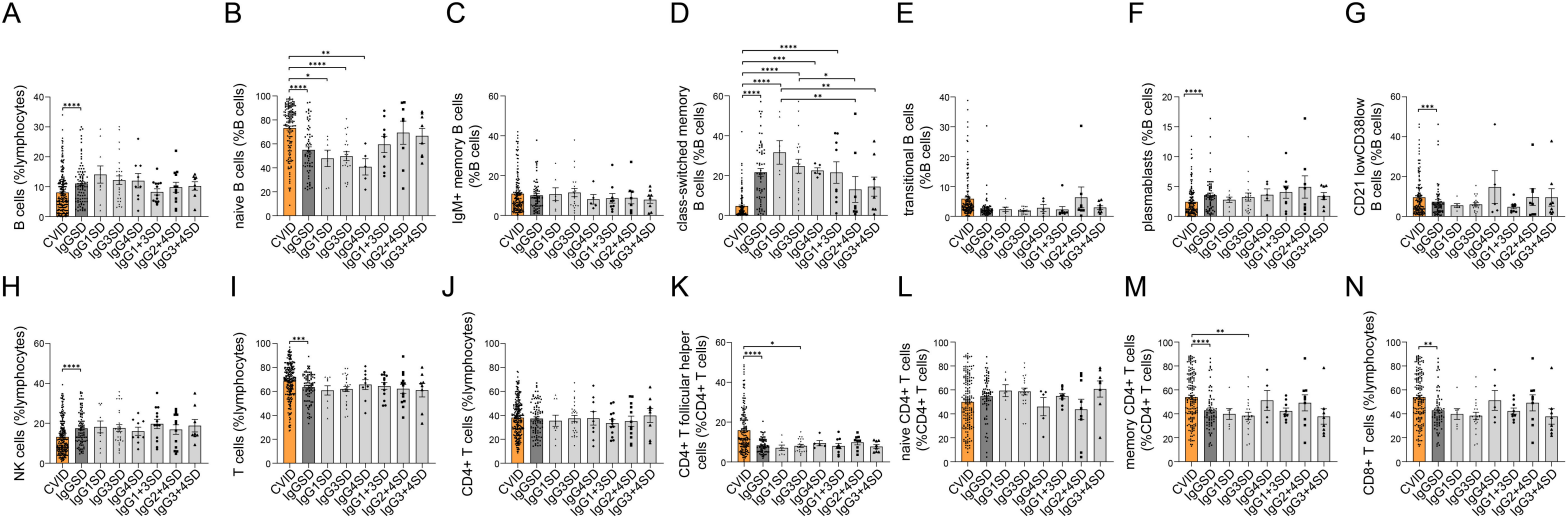
Comparison of lymphocyte subsets between common variable immunodeficiency (CVID) and IgG subclass deficiencies (IgGSDs) as well as across the distinct IgGSDs. Following lymphocyte subsets are shown: B cells **(A)** and B-cell subsets **(B–G)**, NK cells **(H)**, T cells **(I)**, and T-cell subsets **(J–N)** (*p < 0.05, **p < 0.01, ***p < 0.001, and ****p < 0.0001).

A comparison of studied peripheral lymphocyte subset counts between CVID and all IgGSDs revealed significantly lower cumulative percentages of B cells in CVID. Within B cells, we observed significantly higher quotes of naïve B cells and CD21^low^CD38^low^ B cells, accompanied by lower percentages of class-switched memory B cells and plasmablasts in CVID as compared to IgGSDs (**Figures 2A**). In addition, CVID patients displayed significantly lower NK cell counts and higher cumulative T-cell percentages, including higher memory CD4^+^ T cells, CD4+ T follicular helper cells, and CD8^+^ T cells (**Figures 2H**).

When considering each IgGSD separately, the subgroups of patients with IgG1SD, IgG3SD, and IgG4SD displayed significantly lower naïve B-cell percentages as compared to CVID (**Figure 2**). With the exception of IgG2 + 4SD and IgG3 + 4SD, in all other IgGSDs, class-switched memory B-cell percentages were significantly higher than in CVID (**Figure 2**). Furthermore, across the different IgGSDs, class-switched memory B cells were significantly lower in IgG2 + 4SD as compared to IgG1SD and IgG3SD.

## Discussion

IgGSDs had been previously associated with variable immunodeficiency and immune dysregulation (15, 16). However, the identification of IgGSDs in subjects without clinically evident immunological disease questioned the clinical relevance of the diagnosis of IgGSDs. In the present study, similar to the CVID, IgGSDs are associated with recurrent respiratory tract infections and variable forms of autoimmunity. However, despite a considerable phenotypic overlap, identified differences with respect to both the spectrum and severity of infections, as well as the manifestations of immune dysregulation, suggest the differential clinical relevance of the distinct IgGSDs and CVID.

Both IgGSDs and CVID are associated with upper and lower respiratory tract infections, reflecting ineffective humoral immunity. However, respiratory tract infections were more prevalent in CVID than in IgGSDs. The latter and the lower frequency of bronchiectasis and the lower quote of IgGSD patients receiving immunoglobulin replacement are consistent with a milder degree of humoral immunodeficiency in IgGSDs as compared to the CVID. In addition, a comparative analysis of B-cell subsets revealed lower class-switched memory B cells in CVID than in most IgGSDs, suggesting a more profound block in B-cell maturation in CVID, which may account for a more severe humoral immunodeficiency (21).

The reason for the higher rate of recurrent HSV reactivations in IgGSDs remains unclear. Overall, the mechanisms maintaining the latency of HSV are not well understood (22). Possible immunological mechanisms maintaining HSV latency may include innate immune recognition, such as the stimulator of interferon genes (STING) and Toll-like receptor 3 (TLR3) (23, 24). Given the detection of humoral immune responses against HSV across all four IgG subclasses and recent studies revealing the protective effect of maternal antibodies from neonatal infections (25–27), HSV-specific humoral responses may be directly involved in the maintenance of HSV latency. Immune cells that may be involved in the maintenance of HSV latency include NK cells and local, infected neuronal cell-proximal adaptive immune responses, including CD4^+^ T cells and virus-specific CD8^+^ T cells (28, 29), which need to be further investigated in patients with IgGSDs. In particular, defects in innate immune sensing and signaling and consequent aberration in cytokine responses and Th skewing may affect B-cell differentiation and class-switched recombination (30), leading to both IgGSD and impaired immune surveillance of latent HSV infection.

Among IgGSDs, IgG2 + 4SD is associated with the highest rate of lower respiratory tract infections and bronchiectasis. The latter may reflect the role of IgG2 antibodies in immunity against bacteria-producing capsular polysaccharides, such as *Streptococcus pneumoniae*, which commonly cause pneumonia (14, 15). The higher quote of patients with IgG2 + 4SD with lower respiratory tract infections and bronchiectasis and the lower counts of class-switched memory B cells as compared to most other IgGSDs are features resembling CVID. The higher frequency of recurrent mucocutaneous HSV infections in IgGSD and the association of herpes zoster with IgG3SD in the present study come in line with previous studies, suggesting failed control of HSV and varicella zoster virus (VZV) in IgGSDs (31–33). In particular, susceptibility to HSV infections has been associated with lower serum levels of IgG1 and IgG3, while recurrent herpes zoster has been reported in patients with IgG3SD. These associations may suggest a role for IgG1 and IgG3 antibodies in the context of antiviral immune responses. IgG1 and IgG3 antibodies largely represent the humoral immune response to HSV, while in the case of varicella zoster virus, IgG3-specific antiviral antibodies have been reported to dominate (25, 34).

Autoimmune cytopenias, especially ITP, represent the most common autoimmune manifestation of CVID (5, 35). Our results suggest that autoimmune cytopenias are considerably rarer in IgGSDs as compared to CVID, while inflammatory arthritis was significantly more common. The latter findings together with the significantly lower rates of lymphoproliferative manifestations, particularly lymphadenopathy and splenomegaly, in IgGSDs reveal a differential spectrum of immune dysregulation and provide evidence of the distinct pathomechanisms of immune dysregulation in IgGSDs and CVID. Nevertheless, in this case, the higher rate of splenomegaly in IgG2 + 4SD and the fact that ITP in IgGSDs was diagnosed only in the case of two patients with IgG2 + 4SD distinguish this IgGSD from the rest while providing a phenotypic link between IgG2 + 4SD and CVID. In a previous study, Barton et al. performed a comparative analysis of autoimmune conditions in CVID and IgGSDs (16). Barton et al. identified a higher prevalence of Sjögren’s syndrome and hypothyroidism in CVID, deviating from the findings of the current study, while common manifestations of immune dysregulation, such as ITP and splenomegaly, were not evaluated. In the aforementioned study, IgGSDs were also diagnosed in patients with reduced total IgG levels, falling under unclassified antibody deficiency according to current ESID criteria. Furthermore, the aforementioned group of patients was compared to a relatively small cohort of CVID patients, whose clinical features, such as the very high prevalence of Sjögren’s syndrome (diagnosed in 20.6% of patients), deviated from the phenotypic spectrum of CVID in most published cohorts, suggesting a possible selection bias, which may explain the discrepancy with the findings of the present study.

Our study has several limitations owing to its retrospective design. Further, the relatively small number of patients with IgGSDs, especially the rarest ones, hampered their phenotypic evaluation and may have resulted in failure to detect significant disorder-specific differences. The latter highlights the need for multi-center studies, including larger numbers of patients. An additional limitation of our study is the fact that all studied patients were recruited from an outpatient clinic, focusing on immunodeficiency patients, which may have led to the overestimation of infectious manifestations and, overall, the clinical burden of IgGSDs, which have been previously also reported in asymptomatic blood donors. However, in the present work, a comparative evaluation of phenotypes and treatments of patients from the same center, diagnosed according to the current ESID diagnostic criteria, whose phenotypes were documented following the same procedures, reveals the differential clinical significance of IgGSDs as compared to CVID. Our findings show that the clinical spectrum of IgGSDs is different from one of CVID, both with respect to the severity and spectrum of infectious manifestation as well as regarding manifestations of immune dysregulation. Identified differences in peripheral lymphocyte subsets, especially within B cells, suggest the differential pathomechanisms of IgGSDs and CVID. Further, the presented findings may be relevant for the management and follow-up of patients with IgGSDs. In particular, the higher prevalence of herpes zoster in patients with IgG3SD should lead to consideration and timely vaccination with the recombinant zoster vaccine. Furthermore, given the broad phenotypic overlap between IgG2 + 4SD and CVID, we suggest the need for regular immunological monitoring, including the timely consideration of immunoglobulin replacement treatment and diagnostic evaluation for bronchiectasis.

In summary, the relatively high rates of patients with infections and autoimmunity strongly suggest that IgGSDs are clinically relevant PADs, whose phenotypic spectrum overlaps with CVID. However, differences with respect to the spectrum and severity of infections, the manifestations of immune dysregulation, and immunophenotypic findings distinguish most IgGSDs from CVID and suggest a differential pathogenesis.

## Data Availability

The original contributions presented in the study are included in the article/**Supplementary Material**. Further inquiries can be directed to the corresponding author.
