# Supplementary material for: Reappraisal of IgG subclass deficiencies: a retrospective comparative cohort study
Source: Front Immunol. 2025 Apr 17;16:1552513. doi: 10.3389/fimmu.2025.1552513 (PMC12043879; doi:10.3389/fimmu.2025.1552513)
Supplement: Supplementary file 1 [file DataSheet1.pdf]

Supplement

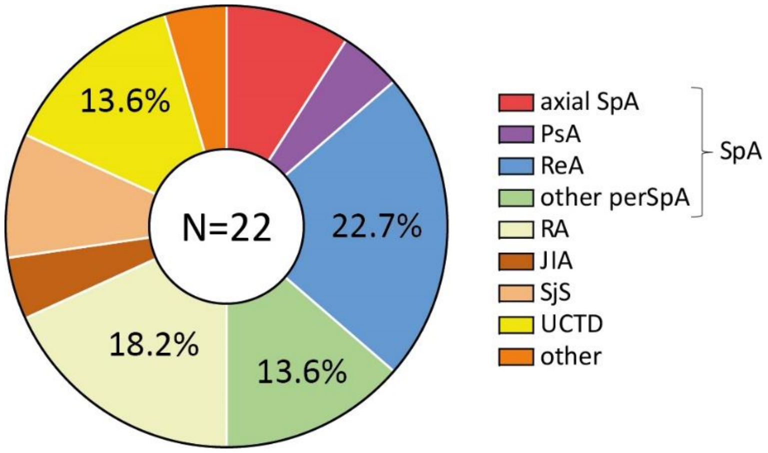

Suppl. Figure 1: Classification of arthritis in 22 patients with IgGSDs (JIA, juvenile idiopathic arthritis; perSpA, peripheral spondyloarthritis; PsA, psoriatic arthritis; RA, rheumatoid arthritis; ReA, reactive arthritis; SjS, Sjögren syndrome; SpA, spondyloarthritis; UTCD, undifferentiated connective tissue disease)

Suppl. Table 1: Frequency of recurrent mucocutaneous herpes simplex infections in each IgG subclass deficiency disorder as well as in patients with low IgG1, IgG2, IgG3 or IgG4 levels.

IgGSD	**recurrent HSV (*N*=17)**	**no history of recurrent HSV (*N*=79)**	**OR**	**95% CI**	***p* value**
IgG1SD (N=11)	0 (0)	11 (13.92)	0	0-1.39	0.2242
IgG3SD (N=30)	5 (29.41)	25 (31.65)	0.9	0.32-2.65	0.9139
IgG4SD (N=9)	3 (17.65)	6 (7.59)	2.61	0.65-9.97	0.4058
IgG1+3SD (N=15)	2 (11.76)	13 (16.46)	0.68	0.14-2.83	0.9084
IgG2+4SD (N=13)	1 (5.88)	12 (15.19)	0.35	0.03-2.33	0.5308
IgG3+4SD (N=8)	2 (11.76)	6 (7.59)	1.62	0.31-7.55	0.9358
Low IgG1 (N=29)	3 (17.65)	26 (32.91)	0.44	0.13-1.54	0.3410
Low IgG2 (N=20)	4 (23.53)	16 (20.25)	1.21	0.39-4.12	0.9781
Low IgG3 (N=58)	11 (64.71)	47 (59.49)	1.29	0.45-3.83	0.9003
Low IgG4 (N=36)	7 (41.18)	29 (36.71)	1.21	0.45-3.36	0.9450

CI, confidence interval; HSV, herpes simplex virus; IgGSD, IgG subclass deficiency; *N*, total number; n, number; OR, odds ratio

Suppl. Table 2: Lymphocyte subsets in patients with or without a history of recurrent mucocutaneous herpes simplex infections.

Lymphocyte subset	**recurrent HSV (*N*=17)**	**no history of recurrent HSV (*N*=79)**	***p* value**
B cells (cells/µl) – median (IQR)	221 (95-437)	140 (86-236)	0.2074
B cells (% lymphocytes) – median (IQR)	10 (5-29)	10.4 (5.9-30)	0.7984
T cells (cells/µl) – median (IQR)	1332 (1099-1469)	984.5 (771-1438)	0.0626
T cells (% lymphocytes) – median (IQR)	71 (58.9-76.3)	65 (54.9-72.2)	0.1563
$CD4^+$ T cells (cells/µl) – median (IQR)	648.5 (500-1109)	534 (404-726)	0.1007
$CD4^+$ T cells (% lymphocytes) – median (IQR)	39.7 (26-45.6)	34.7 (26.7-48.5)	0.7243
$CD8^+$ T cells (cells/µl) – median (IQR)	480 (280-681)	346 (233-506)	0.1096
$CD8^+$ T cells (% lymphocytes) – median (IQR)	28.8 (15.1-36.5)	22 (17.3-29.8)	0.3759
NK cells (cells/µl) – median (IQR)	351 (219-476)	283 (167-396)	0.1896
NK T cells (% lymphocytes) – median (IQR)	17.8 (9.2-21)	16.4 (11.2-24.3)	0.8886
$CD4^+$ follicular helper cells (% $CD4^+$ T cells) – median (IQR)	9.7 (4.5-12.2)	7.4 (5.2-32.7)	0.7814
Naïve $CD4^+$ T cells (% $CD4^+$ T cells) – median (IQR)	57.8 (54.1-64.7)	57.5 (47-67.9)	0.5756
Memory $CD4^+$ T cells (% $CD4^+$ T cells) – median (IQR)	39.6 (32.8-86.4)	38.9 (28.5-53.2)	0.8093

HSV, herpes simplex virus; IQR, interquartile range; *N*, total number
